# A novel bioaugmentation technique effectively increases the skin-associated microbial diversity of captive eastern hellbenders

**DOI:** 10.1093/conphys/coaa040

**Published:** 2020-09-08

**Authors:** Erin K Kenison, Obed Hernández-Gómez, Rod N Williams

**Affiliations:** 1 Department of Forestry and Natural Resources, Purdue University, 715 W. State Street, West Lafayette, IN 47907, USA; 2 Idaho Fish and Wildlife Office, U.S. Fish and Wildlife Service, 1387 S. Vinnell Way, Boise, ID 83706, USA; 3 Department of Natural Sciences and Mathematics, Dominican University of California, 50 Acacia Ave., San Rafael, CA 94901, USA

**Keywords:** alpha diversity, amphibian, captivity, community composition, skin microbiota, translocation

## Abstract

Captive environments are maintained in hygienic ways that lack free-flowing microbes found in animals’ natural environments. As a result, captive animals often have depauperate host-associated microbial communities compared to conspecifics in the wild and may have increased disease susceptibility and reduced immune function. Eastern hellbenders (*Cryptobranchus alleganiensis alleganiensis*) have suffered precipitous population declines over the past few decades. To bolster populations, eastern hellbenders are reared in captivity before being translocated to the wild. However, the absence of natural microbial reservoirs within the captive environment diminishes the diversity of skin-associated bacteria on hellbender skin and may negatively influence their ability to defend against pathogenic species once they are released into the wild. To prepare hellbenders for natural bacteria found in riverine environments, we devised a novel bioaugmentation method to increase the diversity of skin microbial communities within a captive setting. We exposed juvenile hellbenders to increasing amounts of river water over 5 weeks before translocating them to the river. We genetically identified and phylogenetically compared bacteria collected from skin swabs and river water for alpha (community richness) and beta (community composition) diversity estimates. We found that hellbenders exposed to undiluted river water in captivity had higher alpha diversity and distinct differentiation in the community composition on their skin, compared to hellbenders only exposed to well water. We also found strong evidence that hellbender skin microbiota is host-specific rather than environmentally driven and is colonized by rare environmental operational taxonomic units in river water. This technique may increase hellbender translocation success as increasing microbial diversity is often correlated with elevated disease resistance. Future work is necessary to refine our methods, investigate the relationship between microbial diversity and hellbender health and understand how this bioaugmentation technique influences hellbenders’ survival following translocation from captivity into the wild.

## Introduction

Host microbial communities are important for metabolism, vitamin production, resilience to stress and environmental change and immune responses (Turnbaugh *et al.*, 2007; Woodhams *et al.*, 2007; Loudon *et al.*, 2014). Subsequently, a stable and diverse cutaneous microbiome is often positively correlated with host health and resiliency against pathogens (Woodhams *et al.*, 2011; Antwis *et al.*, 2014; Loudon *et al.*, 2014). Amphibians acquire diverse bacteria from their surrounding environment; being in direct contact with bacterial reservoirs can lead to colonization of rare or transient species (Loudon *et al.*, 2014). Disturbances, such as removing eggs or animals from their natural environment and placing them into captivity, can be a major perturbation to amphibian skin microbiota (Redford *et al.*, 2012; Loudon *et al.*, 2014). Captive environments often lack natural microbial reservoirs, have filtration systems that remove microbes and are maintained in hygienic ways to prevent the spread of disease. Thus, individuals brought to, or reared in, captivity quickly lose cutaneous microbe diversity because of aseptic rearing conditions and have little to no opportunity for environmental bacteria to re-colonize their skin (Antwis *et al.*, 2014; Becker *et al.*, 2014; Loudon *et al.*, 2014). This results in significantly different skin microbial diversity among captive amphibians compared to wild conspecifics (e.g. golden frog [*Atelopus zeteki*] and fire-bellied toads [*Bombina orientalis*]; Becker *et al.*, 2014; Bataille *et al.*, 2015).

Without diverse skin microbiota and previous exposure to naturally occurring microorganisms, an individual’s immune system may be underdeveloped and lack specialized responses to natural threats (Magnadóttir, 2006; Richmond *et al.*, 2009). Subsequently, animals in captivity likely have depauperate and atypical skin microbiota and naïve immune systems and may be more susceptible to pathogenic species following introduction to a natural system (Alberts, 2007; Boyce *et al.*, 2011; Loudon *et al.*, 2014; Sabino-Pinto *et al.*, 2016). To increase skin microbial diversity on captive animals, rearing programs are incorporating probiotics, pre-exposure or bioaugmentation strategies (Moriarty, 1999; Irianto and Austin, 2002; Harris *et al.*, 2009; Becker and Harris, 2010; Merrifield *et al.*, 2010; Woodhams *et al.*, 2011). However, these techniques often focus on exposing only one microorganism at a time, are commonly directed toward resistance against the fungus *Batrachochytrium dendrobatidis* and do not always increase host health (e.g. *Atelopus* species; Becker *et al.*, 2012). However, amphibians are naturally exposed to a variety of bacterial, viral and fungal organisms in soil and water; therefore, an environmental reservoir with a suite of microbes (6 × 10^12^ bacterial cells/m^2^ of soil, 10 × 10^5^ bacterial cells/mL of river water; Whitman *et al.*, 1998) would better mimic natural conditions, compared to a single-species probiotic. Although a more complex bioaugmentation method has the potential to unintentionally introduce pathogens, presenting free-flowing microbes in captivity provides the opportunity for amphibian skin microbiota to become diversified by naturally occurring microorganisms prior to release. Furthermore, the safety of captivity allows animals to be monitored and treated if disease symptoms develop.

Captive-rearing efforts are underway to conserve wild populations of eastern hellbenders (*Cryptobranchus alleganiensis alleganiensis*; Ettling *et al.*, 2013; Kenison and Williams, 2018). These large, fully aquatic salamanders historically occurred throughout much of the eastern and central USA (Mayasich and Phillips, 2003). However, over the past few decades, hellbender populations have exhibited precipitous declines and are now listed as threatened or endangered throughout much of their range (Mayasich and Phillips, 2003; Burgmeier *et al.*, 2011). In Indiana, hellbenders are state-endangered and exist only in the Blue River in southern Indiana, making them extremely vulnerable to extirpation (Kern, 1984; Wheeler *et al.*, 2003; Unger *et al.*, 2013). In captivity, hellbenders are reared in homogeneous environments that are nearly devoid of free-flowing microbes present in a riverine environment, thus limiting environmental transmission to the host-associated skin microbiome (Ettling *et al.*, 2013). Furthermore, hellbenders experience parental care by males in the wild (Nickerson and Mays, 1973), which could facilitate vertical transmission of skin microbiota from fathers to their offspring (Walke *et al.*, 2011; Hughey *et al.*, 2017). However, hellbenders are usually removed from the river as eggs before they have been exposed to river or parental microbiota as larvae (O. Hernández-Gómez, personal communication). Subsequently, captive hellbenders have distinctly different microbial communities than wild conspecifics (Hernández-Gómez *et al.*, 2019). In addition, young hellbenders have weaker immune systems than adult hellbenders, making it especially important for them to bolster their skin microbiome before translocation in order to prevent mortalities associated with infectious disease (Bodinof *et al.*, 2012; Hopkins *et al.*, 2016). Without a diverse and stable microbiome, exposure to novel microorganisms has the potential to negatively influence the defence mechanisms of naïve, captive-reared individuals following release into the wild (Hopkins *et al.*, 2016). This becomes a major concern for hellbender augmentation efforts in Indiana, where rapid population losses necessitate maximizing translocation success (Unger *et al.*, 2013).

Translocation programs have been developed to curb declines and augment remaining hellbender populations, but have had varying levels of success (17–72% survival; Bodinof, 2010; Boerner, 2014; Kraus *et al.*, 2017). Hygienic captive-rearing environments and depauperate skin microbial communities may influence the success of these programs (Redford *et al.*, 2012; Becker *et al.*, 2014). We developed a novel form of bioaugmentation to address the dissimilarities in skin microbial communities between captive and wild hellbenders. Our primary goals were to investigate (i) whether exposure to river water in a captive setting could increase microbial diversity on the skin of captive-reared hellbenders, (ii) if communities found on the skin following inoculation resemble those found in river water and (iii) how microbial diversity of pre-exposed and naïve hellbenders change following release into the wild. Exposing hellbenders to river water, and its diverse microbiome, prior to release may be the most effective way to increase their cutaneous defences against potentially pathogenic bacteria and facilitate a successful transition from captivity to the river.

## Materials and methods

We incrementally exposed captive hellbenders to river water to facilitate the colonization of naturally occurring cutaneous microbiota and diversify symbiotic skin microbes in a captive setting, before we released them into their natal Indiana river. We altered the biotic environment in their tanks each week for 5 weeks to more closely mimic natural, riverine conditions in captivity. At the end of each experimental week, we collected skin microbiota samples to assess changes in alpha (within community diversity) and beta (community composition) diversity (Redford *et al.*, 2012; Becker *et al.*, 2014; Jiménez and Sommer, 2016). We also collected river water samples to compare similarities between hellbender skin and the riverine microbiota. Following the 5-week experimental period in captivity, we released all hellbenders for 7 days into cages within the river and again collected skin microbiota: this totalled six sampling weeks between captivity and the field. All animals were handled following procedures approved through Purdue University’s Animal Care and Use Committee [protocol #1406001094].

### Experimental design

The hellbenders used for this study were collected as eggs from the Blue River in southern Indiana in 2013 and were reared at Purdue University’s Aquaculture Research Laboratory without any exposure to Blue River water until the start of this experiment—3 years after hatching. On 21 June 2016, we haphazardly chose 36 3-year-old hellbenders for this study and randomly assigned them to six separate 132-L tanks (six hellbenders per tank). We housed all tanks on a two-tiered rack in a temperature-controlled room (14°C ± 1°C). We provided hellbenders with tile hides as refugia and black worms (*Lumbriculus variegatus*) every 3 days as food. Half of the individuals were control individuals and only received UV-treated and filtered well water (*n* = 18) while treatment individuals (*n* = 18) received supplementary river water. We placed all treatment tanks on the bottom rack, with control tanks stacked above to avoid inadvertent river water contamination. We also used separate nets and equipment between treatment and control tanks to reduce cross contamination between groups. We waited 1 week before adding river water, in order to collect baseline skin microbiota data as well as allow hellbenders to acclimate to their rearing tanks. We added 100 mL of river water to treatment tanks during the second week and increased the amount by an order of magnitude every week thereafter (100 mL (0.1% river water concentration), 1 L (1%), 10 L (10%), and then 100 L (100%), respectively). Following the addition of river (treatment tanks) or filtered well (control tanks) water, we maintained static conditions within all tanks for 7-day exposure periods. Previous studies have successfully inoculated amphibians with a probiotic bath for 2–48 h; therefore, week-long exposures should provide ample time for bacterial colonization (Harris *et al.*, 2009; Vrendenburg *et al.*, 2011; Bletz *et al.*, 2013). We retrieved new Blue River water for treatment tanks at the beginning of Weeks 2 through 5. We collected water in five gallon buckets, drove the water 3 h to Purdue University in coolers, and then added it directly to treatment tanks—all buckets and coolers were cleaned and dried before river water was collected. We conducted complete water changes for all treatment and control tanks at the beginning of each week.

After the last lab-sampling event (Week 5), we transported hellbenders (see Kenison *et al.*, 2016) to the Blue River in southern Indiana. Treatment hellbenders were placed together in one cooler of the transport system, but were separated from control individuals for the duration of travel to the river. We placed all 36 hellbenders (18 treatment and 18 controls) into four hardware mesh cages (1′ × 3′ × 3′) (nine hellbenders per cage), being sure to separate hellbenders by treatment and control groups, with tile hides for refuge, closed them securely, and let them remain in the river for 7 days. The cages allowed us to collect hellbenders at the end of the experiment for one last sampling event rather than permanently releasing them into the river.

### Microbiota sampling and processing

We swabbed the dorsum of each hellbender at the beginning of the experiment (28 June 2016) and every week thereafter (6 July through 5 August 2016) to collect skin-associated microbiota (similar to Loudon *et al.*, 2014). We handled hellbenders and sampled their microbiota following the protocol of Hernández-Gómez *et al*. (2017b), being sure to change gloves between individuals, rinse each hellbender with 1 L of autoclaved water, and rub sterile cotton-tipped swabs (Medline Industries Inc., Mundelein, IL) along their dorsum for 30 s. We stored the sample swabs in 1.5-mL microcentrifuge tubes. When we collected swabs in captivity, we immediately placed tubes in a −20°C freezer and moved them on ice to a −80°C freezer within 2 h of collection. In the field, we stored swab tubes in liquid nitrogen and placed them in a −80°C freezer upon return to the laboratory within 24 h of collection.

During each sampling event, we collected 1 L of water from each of the control (*n* = 6) and treatment tanks (*n* = 6) after swabbing and 2 L of river water directly from the Blue River. We stored water samples in a cooler and filtered them in an aseptic environment within 12 h of collection using a Whatman 1.5-μM glass microfiber filter (GE Healthcare, Chicago, IL). We stored all filters in 15-mL centrifuge tubes and placed them in a −80°C freezer until we isolated the DNA.

We extracted DNA from skin swabs using a PowerSoil DNA Isolation Kit (MoBio Laboratories Inc., Carlsbad, CA), following the modified protocol of Hernández-Gómez *et al.* (2017b). We also used the PowerWater DNA Isolation Kit to extract DNA from water filters using the manufacturer’s instructions for organisms difficult to lyse. We used the primer set 27F/338R to amplify the 16S rRNA gene V2 region. We ran all samples in triplicate, each with 25 μL of reaction volume: 5 μL template DNA, 1× MyTaq Master Mix (Bioline, Taunton, MA), 0.7 μM of forward and reverse primers and 6.5 μL of water (MoBio Laboratories, Inc., Carlsbad, CA). We ran the PCRs with 2 min at 95°C, 30 cycles for 45 s at 94°C, 60 s at 50°C, 90 s at 72°C and 10 min at 72°C. We pooled the triplicates from each sample, cleaned the PCR products with an UltraClean PCR Clean-up kit (MoBio Laboratories, Inc., Carlsbad, CA), and then performed a second PCR. Our second PCR was used to prepare the sequencing library; we added on dual-index barcodes connected to Illumina sequencing adaptors to the end of amplicons (Hernández-Gómez *et al.*, 2017b). We ran each PCR sample with 15 μL of reaction volume: 5 μL clean amplicons, 1× MyTaq Master Mix (Bioline, Taunton, MA), 0.7 μM of forward and reverse barcode primers and 1.5 μL of water (MoBio Laboratories, Inc., Carlsbad, CA). We ran the second PCR with 2 min at 95°C, 5 cycles for 45 s at 94°C, 60 s at 65°C, 90 s at 72°C and 10 min at 72°C. We measured the DNA concentrations of our second PCR products using a Qubit Fluorometer 2.0 (Invitrogen Corp, Carlsbad, CA) and pooled our samples in equimolar amounts. We used the Reagent Kit V2 on a MiSeq machine (Illumina, Inc., San Diego, CA) at the Purdue Genomics Core Facility to sequence the sample pool and produce 250 bp paired-end reads.

We processed raw sequence reads, assigned operational taxonomic units and generated species abundance tables for all skin swabs and water samples following Hernández-Gómez *et al.* (2017a). In brief, we implemented custom Python programs developed by Hernández-Gómez *et al.* (2017b) and the Quantitative Insights Into Microbial Ecology (QIIME) version 1.9.1 pipeline (Caporaso *et al.*, 2011) to filter erroneous reads, cluster reads into operational taxonomic units (OTUs) and generate abundance based OTU tables using the open-open reference protocol (Rideout *et al.*, 2014). To limit the inclusion of any OTUs derived from sequencing errors, such as base miscalls or chimeras, we filtered out OTUs that represented <0.005% of the total read count (Bokulich *et al.*, 2013). To standardize read depth across all samples we rarefied the OTU table to 1699 reads per sample. We chose the 1699 read cut-off in order to maximize the number of samples included in further analyses. We ran all further analyses at a greater rarefaction cut-off of 5000 reads per sample and did not observe differences in the conclusions derived from either sequencing depth.

## Statistical analyses

We ran all analyses in program R version 3.3.2 (R Core Team, 2016). We investigated differences in skin microbiota between treatment and control hellbenders across the 5 weeks in captivity and after release into the river at Week 6. We assessed differences in skin microbiota community richness (observed OTUs) and community diversity (Shannon diversity indices—a species abundance–weighted metric), both of which were calculated in QIIME using the relative-abundance–based OTU table. We used generalized linear mixed-effects models assuming a Gaussian distribution to compare treatment and control groups at each of the six experimental weeks, while accounting for repeated measures. We did not identify each hellbender prior to swabbing; therefore, we assigned ‘rearing tank’ as our random effect. We included ‘week’ and ‘treatment’ as our fixed effects and tested for two-way interactions between ‘treatment’ and ‘week’. We used the package ‘nlme’ for mixed effects analyses and report estimates with standard errors.

We tested for differences in skin microbiota community composition (beta diversity) by comparing phylogenetic-based differences of OTUs between groups. We used the Newick phylogenetic tree with the OTU table in program R to create unweighted UniFrac matrix using the package ‘GUniFrac’. Unweighted distance matrices take into account the presence/absence of observed OTUs and incorporate phylogenetic distances (Lozupone and Knight, 2005). Additionally, we used the package ‘vegan’ to generate a non-phylogenetic dissimilarity metric, Bray–Curtis, which accounts for differences in OTU abundance among samples. We performed Anosim tests (package ‘vegan’) using unweighted UniFrac and Bray–Curtis distances to partition the variation between control and treatment groups. We visualized differences in community composition between control and treatment groups at each week and community composition across weeks by plotting principle coordinate analyses (PCoAs) using unweighted UniFrac and Bray–Curtis distances (package ‘phyloseq’). We also compared the proportion of shared OTUs between control and treatment hellbenders to identify similarities between groups. We conducted a linear regression with a Gaussian distribution to detect changes in the proportion of shared OTUs through time. Lastly, we calculated the core microbiome (OTUs present in 80% of samples) for each ‘week’ by ‘treatment’ group combination. We report percent differences and model estimates with standard errors.

We also compared the microbial communities found on hellbenders to river water. We compared the proportion OTU control and treatment individuals shared with their tank water and with river water. For each individual, we calculated the proportion of OTUs that were shared with corresponding environmental samples (river water or tank water) by dividing the number of overlapping OTUs by individual OTU richness. We conducted linear regressions to test for differences in the proportion of OTU treatment individuals shared with river water samples through time.

## Results

After filtering out 16S rRNA V4 amplicon sequence reads by base pair quality and length, we processed 3, 293, 935 reads (7–25, 422 reads per sample) using QIIME to produce 1464 OTUs following contaminant removal and rarefaction. Raw sequence data for skin and environmental microbiota samples can be accessed via the BioProject accession number PRJNA474383 in the NCBI Sequence Read Archive database (https://www.ncbi.nlm.nih.gov/sra/).

**Figure 1:**
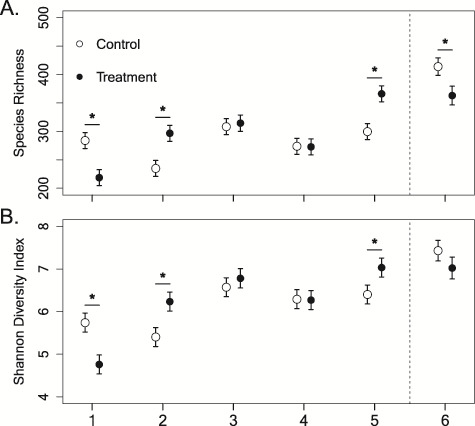
Alpha diversity comparisons including community richness (**A**) and Shannon diversity (**B**) of hellbender (*Cryptobranchus a. alleganiensis*) skin microbiota from control (*n* = 18) and treatment individuals (*n* = 18) over the 5-week experimental period in captivity and following caged release into the wild (Week 6). Treatment individuals were exposed to increasing amounts of river water from Weeks 2 through 5 and were exposed to undiluted river water at Week 5. There was some unresolved variability in Weeks 1 and 2, but notable changes were seen at Week 5 when undiluted river water was provided to treatment hellbenders. All skin swab samples were taken between 28 June and 5 August 2016

**Figure 2:**
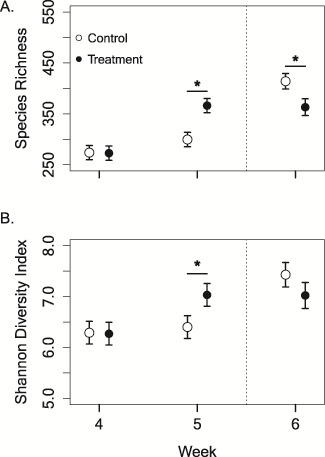
Alpha diversity comparisons, community richness (**A**) and Shannon diversity (**B**), of hellbender (*Cryptobranchus a. alleganiensis*) skin microbiota from control (*n* = 18) and treatment individuals (*n* = 18) during Weeks 4 and 5 in captivity, and Week 6 following river release. Treatment individuals were exposed to 10% river water in Week four, undiluted river water at Week 5, and all individuals were placed in the river for 7 days before Week 6 sampling. The dotted line indicates when all control and treatment hellbenders were released into the river. Treatment hellbenders had a large increase in richness and diversity between Weeks 4 and 5, but no change between Weeks 5 and 6. Control individuals did not differ between Weeks 4 and 5, but had a rapid increase in community richness and Shannon diversity following river release. All skin swab samples were taken between 22 July and 5 August 2016. Estimates are back-transformed and presented with standard error bars. Asterisks denote significant (*α* < 0.05) differences between control and treatment individuals

We found evidence for differences in community richness on hellbender skin across the 6-week experimental period in captivity that depended on treatment group (Fig. 1). There was variability in community richness for the first 2 weeks of the experiment. In Week 1, treatment individuals had 32% less richness than control individuals (estimated difference = 65.3, *t* value = 3.29, *P* = 0.001), but in Week 2, they had 21% greater richness (estimated difference = 61.9, *t* value = 3.1, *P* = 0.002). Species richness was comparable between treatment and control individuals in Weeks 3 and 4 (*P* > 0.05), yet in Week 5, when treatment hellbenders were exposed to undiluted river water, they had 22% greater richness than control individuals (estimated difference = 66.6 OTUs, *t* value = 3.35, *P* = 0.001; Fig. 2). Treatment individuals had a 25% increase in community richness from Weeks 4 to 5, but no change in richness between Weeks 5 and 6 (Fig. 2). Control individuals had a 28% increase in community richness between Week 5 in captivity and Week 6 in the river, which was similar to treatment individuals between Weeks 4 and 5 (Fig. 2). In Week 6, following caged release into the river, treatment individuals had 15% fewer OTUs compared to control individuals (estimated difference = 51.0 OTUs, *t* value = 2.54, *P* = 0.012).

We found trends in Shannon diversity were similar to those in community richness through time (Fig. 1). In Week 1, treatment individuals had 22% lower Shannon diversity compared to control individuals (estimated difference = 0.98, *t* value = 3.12, *P* = 0.002). However, by Week 2, treatment individuals inoculated with low levels of river water had 15% greater diversity on their skin (estimated difference = 0.83, *t* value = 2.64, *P* = 0.009). Weeks 3 and 4 were comparable between treatment and control individuals, but treatment individuals had 10% greater diversity than control individuals by Week 5 (estimated difference = 0.63, *t* value = 2.01, *P* = 0.046; Fig. 2). After caged release into the river, both treatment and control individuals had similar Shannon diversity on their skin (*P* = 0.167; Fig. 2).

**Figure 3:**
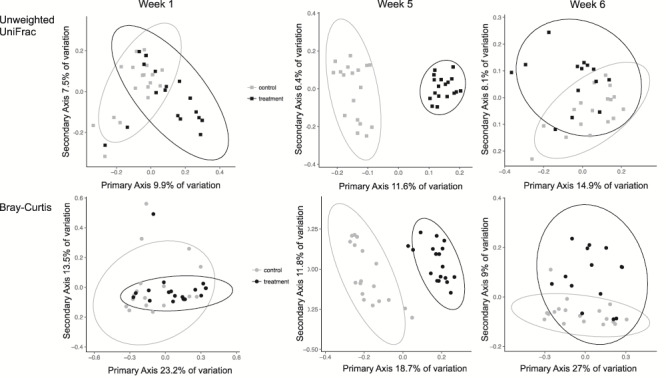
PCoA plots depicting beta diversity comparisons between treatment and control hellbenders (*Cryptobranchus a. alleganiensis*) at Weeks 1, 5 and 6. Gray points depict control individuals (squares = unweighted UniFrac, circles = Bray–Curtis), and black points indicate treatment individuals (squares = unweighted UniFrac, circles = Bray–Curtis). Ellipses illustrate overlap in community composition between the two hellbender groups. There is strong overlap between control and treatment individuals at the beginning of the experiment and after all hellbenders are released into the river (Weeks 1 and 6). However, there is a strong differentiation between groups at Week 5, when treatment hellbenders are bathed in undiluted river water

**Figure 4:**
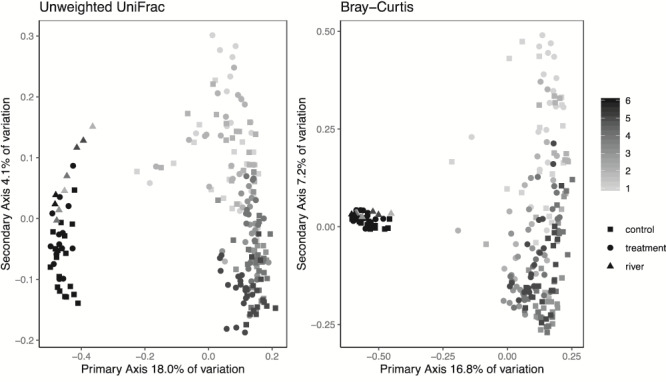
PCoA plots depicting beta diversity comparisons of microbiota on the skin of treatment and control hellbenders (*Cryptobranchus a. alleganiensis*) and river water over the 6-week experimental period. The color gradient is indicative of experimental week, with Week 1 being depicted as light gray and Week 6 being depicted as black. Square points indicate control individuals, circular points are used for treatment individuals and triangular points correspond to Blue River water samples. We have presented unweighed UniFrac and Bray–Curtis methods, both of which show a distinct separation in beta diversity at Week 6 compared to Weeks 1–5, regardless of group and overlap in composition between hellbender skin at Week 6 and all river water samples

We found distinct differentiation in community composition measurements between control and treatment hellbenders at each of the experimental weeks (Table 1). PCoA plots demonstrated overlapping community composition at Week 1, but distinct separation between groups at Week 5 (Fig. 3). In Week 5, the variation accounted for by treatment was nearly twice as much as in Week 1 (Week 1: Anosim *R* = 0.314; Week 5: Anosim *R* = 0.77 for unweighted UniFrac, Table 1). Following caged release into the river, control and treatment hellbenders had overlapping microbial communities and little variation explained by group (Week 6: Anosim *R* = 0.162 for unweighted UniFrac; Fig. 3). Furthermore, the skin of treatment and control individuals overlapped closest with water samples at Week 6 (Fig. 4). We also found significant differences in beta diversity across all 6 weeks combined, with week explaining the most variation (unweighted Anosim *R* = 0.642, *P* = 0.001, Bray–Curtis Anosim *R* = 0.692, *P* = 0.001). PCoA plots demonstrated that the largest separation in community composition across weeks occurred between Week 6 and Weeks 1 through 5 (Fig. 4).

**Table 1:** Anosim test results for differences between beta diversity measures of the community composition of bacterial species on the skin of treatment and control individuals

Week	Beta diversity test	Anosim *R*	*P* value
1	Unweighted	0.314	0.001
	Bray-Curtis	0.187	0.001
2	Unweighted	0.561	0.001
	Bray–Curtis	0.392	0.001
3	Unweighted	0.52	0.001
	Bray–Curtis	0.664	0.001
4	Unweighted	0.619	0.001
	Bray–Curtis	0.472	0.001
5	Unweighted	0.772	0.001
	Bray–Curtis	0.666	0.001
6	Unweighted	0.162	0.005
	Bray–Curtis	0.126	0.024

Beta diversity tests were conducted with unweighted UniFrac and Bray–Curtis distance matrices. The *R* values indicate the amount of variation explained by treatment

**Figure 5:**
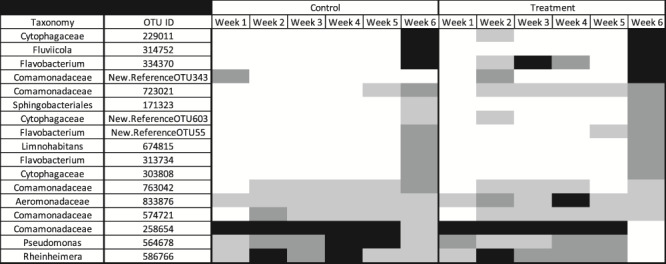
Heatmap of core bacteria (present in 80% of samples) within control (C) and treatment (T) hellbenders (*Cryptobranchus a. alleganiensis)* . The frequencies of core OTUs are presented across the six experimental weeks. Color corresponds with relative abundances >2% (black), between 0.2 and 2% (dark gray), between 0 and 0.2% (light gray) and 0 (white). Table is truncated to show only OTUs with a relative abundance >0.1% at any time point

The size of the skin core microbiomes for each ‘treatment’ and ‘week’ combination ranged from 33 to 86 OTUs. Prior to river release, we observed multiple OTUs present on both treatment and control hellbenders’ skin (Weeks 1–5; Fig. 5). For example, OTUs 258654 (taxonomy: Comamonadaceae), New.ReferenceOTU292 (*Flavobacterium* sp.), and New.ReferenceOTU650 (*Flavobacterium* sp.) were abundant in captivity at relative abundances >2%. Following release, we observed an almost complete wipeout of the captivity abundant OTUs. Instead, OTUs 229011 (Cytophagaceae), 314752 (*Fluviicola* sp.), and 334 370 (*Flavobacterium* sp.) became the most abundant taxa (relative abundance >2%) in the skin microbiota of all hellbenders at Week 6. Two of these river-associated OTUs (229011 and 334370) became successfully incorporated into the core microbiome of treatment individuals prior to their release (Fig. 5). Although there was a large shift in the core microbiome following the release into the river, we argue that bioaugmentation with river water successfully provided a reservoir for natural bacteria to colonize treatment hellbenders’ skin in captivity (Fig. 5).

**Figure 6:**
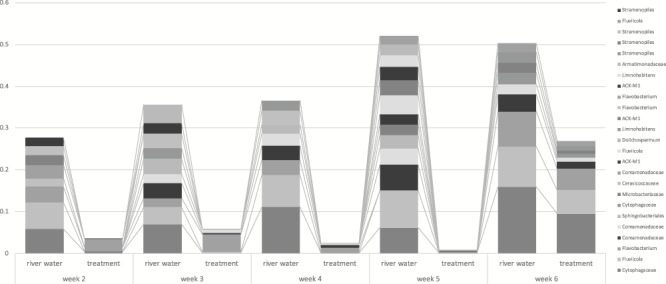
The most common OTUs, their lowest-assigned taxonomy and their relative abundance on river samples and the skin of treatment hellbenders (*Cryptobranchus a. alleganiensis*). Common Blue River water OTUs are found sparingly on hellbender skin, and they do not tend to become relatively abundant until Week 6 after hellbenders had been placed directly into the river for 7 days

In general, we did not observe strong similarities between the OTUs found on the skin of treatment individuals and the OTUs found in river water. Of the most abundant OTUs in river water, we found low relative abundances on the skin of treatment individuals, especially during the first 5 weeks in captivity (Fig. 6). This trend did not change significantly as we incrementally supplemented more river water into treatment tanks (estimated change per week = −22.9 OTUs, *t* value = −0.84, *P* = 0.489). Between Weeks 2 and 4, treatment individuals shared on average 18.9% of their OTUs with river water and control individuals shared 15.4% of their OTUs with river water. This pattern was maintained following exposure to undiluted river water in Week 5 with treatment individuals sharing more OTUs with river water compared to control individuals (14.1% OTUs versus 10.4% OTUs, respectively). Only after release into the river did abundant river water OTUs become more prevalent on hellbenders’ skin (Fig. 6).

## Discussion

We developed a novel environmental inoculation technique that increased hellbender skin microbial community richness and diversity in captivity. Few studies have investigated the use of environmental reservoirs to increase alpha diversity, yet we provide clear evidence that inoculating hellbenders with river water, for 5 weeks, leads to a more specious microbial skin community in a captive setting. Furthermore, undiluted river water was an effective reservoir for a number of natal river bacteria. We observed the largest increase in alpha diversity on the skin of treatment hellbenders following exposure to undiluted river water (100% river water, Week 5) and this change remained even after hellbenders were released into the river (Week 6). Being in constant and complete contact with an environmental reservoir quickly facilitated the immigration and colonization of new species on hellbender skin prior to release, while in the safety of captivity. Eastern red-backed salamanders (*Plethodon cinereus*) that are in constant presence of a soil bacterial reservoir receive environmental transmission, maintain stable alpha diversity and show differences in beta diversity after just 7 days of exposure (Muletz *et al.*, 2012; Loudon *et al.*, 2014). We cannot reconcile the alpha diversity differences detected between control and treatment hellbenders in Week 1; perhaps this was an artifact of randomly selected individuals placed into the control group or an unexpected outcome of changing hellbenders from a dynamic flow-through system to a static system. It is important to note that we found overlapping community composition (i.e. beta diversity) at Week 1, which suggests that differences we saw may have been due to random noise rather than a function of treatment. Furthermore, we found distinct separation in community composition and alpha diversity between treatment and control groups in tandem at Week 5. This demonstrates that environmental reservoirs in captivity (e.g. undiluted river water) support the immigration of new bacteria and the establishment of numerous, potentially beneficial, species on the skin of amphibians (Loudon *et al.*, 2014).

Increasing community richness prior to release into the wild may increase the stability of the skin microbiome. Community stability on the skin has been correlated with increased protection against disturbances (i.e. infections, disease, exposure to new microbiota), because the skin is more likely to harbor communities with increased functional complexity (Hernández-Gómez *et al.*, 2019). However, some studies suggest that major shifts in microbial community composition can be associated with ill health, and higher diversity may lead to more interacting species that could have destabilizing effects on the microbial community (Coyte *et al.*, 2015; Johnson and Burnet, 2016). For example, previous work comparing the skin microbiotas of captive and wild hellbenders has shown that captive skin communities were functionally depauperate and specialized for life in the captive environment (e.g. increased representation of xenobiotic degradation genes; Hernández-Gómez *et al.*, 2019). As such, it is possible that microbiotas on control hellbenders experienced a major disruption following release, which may have facilitated the establishment of opportunistic and potentially pathogenic species (Hernández-Gómez *et al.*, 2017b). Alternatively, the establishment of river bacteria on the skin of treatment hellbenders prior to release may have beneficially influenced the assembly of communities following release (e.g. priority effects; Wuerthner *et al*., *2019*). For example, two river-associated OTUs (229011 and 334370) became successfully incorporated into the core microbiome of treatment individuals prior to their release. We interpret these changes as support for our bioagmentation method; however, we cannot confidently conclude that the jump in species richness on treatment hellbenders’ skin after exposure to undiluted river water is associated with increased stability. Nor can we confirm that the rapid increase in control hellbenders’ alpha diversity following release is evidence of ill health. Future work should evaluate whether repeated exposures to naturally occurring microbiota in deliberate concentrations increases the stability of amphibian microbiomes prior to being exposed to free-flowing microbiota in the wild. Skin microbiota should also be compared for multiple weeks after release in order to understand how inoculations in captivity affect community change through time.

Inoculation was successful; however, differentiations between skin bacterial communities in captivity and following river release suggest our river water reservoirs need to more closely match the microbial composition of a natural, riverine environment. We detected strong differences in the community composition of skin microbiota before and after translocation, regardless of treatment. This suggests that although undiluted river water is proficient at inoculating hellbenders, there is something distinct about the wild environment that is not fully transferred to or maintained in captivity. Although a river’s bacterial community is diverse, the species on hellbender skin may be dependent on what hellbenders are interacting with rather than the water alone (e.g. rocks, substrate, other organisms such as fish or mussels; Whitman *et al.*, 1998; Walke *et al.*, 2014; Rebollar *et al.*, 2016). For example, the OTUs identified to the genera *Flavobacterium*, *Fluviicola* and the family Cytophagaceae were detected in high relative abundance on control and treatment hellbenders only after they were released into the river. These taxa have been identified on the skin of wild hellbenders and must therefore be abundant in or specific to their wild environment (Hernández-Gómez *et al.*, 2017a). Thus, there must be some sources of bacteria in the wild that cannot thrive or be cultivated in captivity, or more simply, were missed during river water collection (Becker *et al.*, 2014). Bacterial species initially present in experimental treatment water, transported from the Blue River, may have also died during the 7-day bathing period. Rearing tanks were maintained with static water rather than a flow-through continually moving design, which may have selected for bacterial species that can thrive in stagnant, lentic environments rather than those reliant on lotic systems. Once bacteria are lost, for any variety of reasons, they become less and less common without a fresh reservoir for replenishment, and are thus less likely to colonize skin (Muletz *et al.*, 2012). Furthermore, more rare species are often at a higher risk of loss during stochastic events, such as temperature change or amphibian skin sloughing (Meyer *et al.*, 2012; Bletz *et al.*, 2013; Loudon *et al.*, 2014). Therefore, bacteria may have colonized hellbender skin during the first 48 h of inoculation, but were lost or unable to recolonize by the time we collected skin swabs.

Even with some dissimilarity between our inoculation environment and the river, we found that captive hellbenders had similar core microbial skin communities to wild con- and hetero-specifics. Wild-captured eastern hellbenders have core OTUs composed primarily of Proteobacteria, Actinobacteria, Firmicutes, Cyanobacteria and Verrucomicrobia (Hernández-Gómez *et al.*, 2017a). The most abundant phylum on the skin of control and treatment hellbenders was Proteobacteria, followed by Actinobacteria, Bacteroidetes, Acidobacteria and Firmicutes. The similarities in the most abundant phyla between wild and captive hellbenders demonstrate that hellbenders have a core microbiota on their skin that is host-specific and that remains comparable under variable conditions. Across multiple amphibian taxa, host species identity is a significant predictor of skin microbial community compared to other factors such as location and environmental variation (McKenzie *et al.*, 2012; Kueneman *et al.*, 2014). We found control and treatment individuals to have the most shared OTUs with each other, regardless of the experimental week. The similarities in skin bacterial species may have been driven by the fact that control and treatment hellbenders likely cohabited in the same tank prior to the beginning of this experiment. All hellbenders would have been reared together after they first hatched, which is a critical time for microbial establishment (Bletz *et al.*, 2013; Warne *et al.*, 2017). Furthermore, all individuals from treatment and control groups were half, if not full, siblings. Hellbenders commonly lie on or beside one another, share tile hides and interact with each other during feeding, all of which may have facilitated horizontal transmission between conspecifics (Moran and Dunbar, 2006). Furthermore, all hellbenders were fed the same black worm (*Lumbriculus variegatus*) diet, which could lead to similarities in skin microbiome across the two experimental groups.

Even with similarities between control and treatment hellbenders, we found clear evidence that amphibian microbial skin communities do not always match the communities of the environment they are in (Kueneman *et al.*, 2014; Walke *et al.*, 2014; Sabino-Pinto *et al.*, 2016). Treatment hellbenders had very few OTUs shared with river water. Of the most dominant OTUs among treatment individuals, some were not even found in river water. Additionally, of the most abundant OTUs found within river water, very few had a relative abundance >0.2% on the skin of hellbenders exposed to undiluted river water. Treatment hellbenders only shared 13% of their OTUs with river water while in captivity. Similarly, wild hellbenders that are continually exposed to river water only share 16% of their OTUs with the river environment (Hernández-Gómez *et al.*, 2017a). Similar trends in environmental-host microbial patterns are seen among bullfrogs (*Rana catesbeiana*) and newts (*Notophthalmus viridescens*); their skin communities harbour OTUs that are generally in low abundance in the environment and the more abundant environmental OTUs are in low abundance on their skin (Walke *et al.*, 2014). This further supports the hypothesis that amphibian skin is not simply a result of their environment, and instead corroborates that hellbender skin is colonized by rare, rather than abundant, environmental microbes (Walke *et al.*, 2014).

Our form of river water bioaugmentation exposed captive hellbenders to natural microbes in a more complex design than usual, single-species probiotic methods. This method allowed us to inoculate multiple hellbenders at once, it required no additional expense in terms of probiotic testing and selection and it produced changes in alpha and beta diversity on the skin of captive hellbenders. Captive hellbenders are at a disadvantage being reared in an aseptic environment. Not only do they lack access to rare environmental bacteria, but their opportunity to acquire skin microbiota through vertical transmission is also eliminated when eggs hatch in captivity. Early life disruptions, like a transfer to captivity, can have profound effects on adult health and disease susceptibility (Knutie *et al.*, 2017). However, we found river water exposure altered hellbenders’ microbial skin community in a way that increased the diversity of skin microbial communities in captivity. With future exploration and refinement, our novel methodology may be able to remedy skin microbiome perturbations and alter hygienic rearing environments in ways that could better prepare hellbenders for translocation into the wild.

If prior exposure to riverine microbiota can have beneficial effects on hellbender health and survival following release, then this method could have positive effects on future translocation projects. Future studies are necessary to refine our methods and investigate ways to optimize this technique in the safety of captivity. Our 7-day exposure time with undiluted water was effective, but exposure in earlier life stages, with longer exposure periods, more frequent water changes or incorporation of river substrate into the captive environment could further increase alpha diversity and make the skin microbiota of captive individuals more similar to that of conspecifics in the wild. Although we did not measure disease susceptibility because of hellbenders’ conservation status, investigating disease dynamics and survival following permanent release into the river could provide more information about the relationship between microbial diversity and disease resistance for hellbenders, and quantify the benefits this technique may have on translocation efforts. Although riverine bacteria are able to quickly colonize the skin of naïve hellbenders following release, prior exposure to free-flowing microbiota in the safety of captivity may be the most feasible way to develop strong cutaneous defences and prime juvenile hellbenders’ immune systems before they are moved from captivity to the wild environment. We suggest river water inoculation methods be further explored in hellbender rearing programs so that we can better understand the long-term effects, both positive and negative, and broad-scale impacts of this technique.
